# Cric searchable image database as a public platform for conventional pap smear cytology data

**DOI:** 10.1038/s41597-021-00933-8

**Published:** 2021-06-10

**Authors:** Mariana T. Rezende, Raniere Silva, Fagner de O. Bernardo, Alessandra H. G. Tobias, Paulo H. C. Oliveira, Tales M. Machado, Caio S. Costa, Fatima N. S. Medeiros, Daniela M. Ushizima, Claudia M. Carneiro, Andrea G. C. Bianchi

**Affiliations:** 1 Graduate Program in Biotechnology, Biological Sciences Research Center (NUPEB), Federal University of Ouro Preto, Ouro Preto, MG Brazil; 2 Cytology Laboratory, Clinical Analysis Department, Federal University of Ouro Preto, Ouro Preto, MG Brazil; 3 Computing Department, Federal University of Ouro Preto, Ouro Preto, MG Brazil; 4 Teleinformatics Engineering Department, Federal University of Ceará, Fortaleza, CE Brazil; 5 Computational Research Division, Lawrence Berkeley National Laboratory, Berkeley, CA USA; 6 Bakar Computational Health Sciences Institute, University of California San Francisco, San Francisco, CA USA; 7 Berkeley Institute for Data Science, University of California Berkeley, Berkeley, CA USA

**Keywords:** Preventive medicine, Population screening, Cancer screening, Biotechnology, Databases

## Abstract

Amidst the current health crisis and social distancing, telemedicine has become an important part of mainstream of healthcare, and building and deploying computational tools to support screening more efficiently is an increasing medical priority. The early identification of cervical cancer precursor lesions by Pap smear test can identify candidates for subsequent treatment. However, one of the main challenges is the accuracy of the conventional method, often subject to high rates of false negative. While machine learning has been highlighted to reduce the limitations of the test, the absence of high-quality curated datasets has prevented strategies development to improve cervical cancer screening. The Center for Recognition and Inspection of Cells (CRIC) platform enables the creation of CRIC Cervix collection, currently with 400 images (1,376 × 1,020 pixels) curated from conventional Pap smears, with manual classification of 11,534 cells. This collection has the potential to advance current efforts in training and testing machine learning algorithms for the automation of tasks as part of the cytopathological analysis in the routine work of laboratories.

## Introduction

Cervical cancer is one of the most frequently diagnosed neoplasms and one of the main causes of death from cancer in the female population, and constitutes a significant public health problem worldwide^1^. According to the most recent estimate by the World Health Organization (WHO), it is the fourth most incident cancer among women worldwide, with approximately 342,000 deaths in 2020, and is the leading cause of cancer death in 42 countries^2^.

Cervical cancer has one of the best prognosis for prevention and cure, reaching almost 100% of cure when diagnosed early with screening methods^3^. The cytopathological procedure known as Pap smear^4^, which is the most widely used test, is relatively inexpensive to perform, and effective in detecting precursor lesions^4^.

The discovery that cervical infection by high oncogenic risk human papillomavirus (HPV) genotypes can progress to cervical cancer has led to the advancement of HPV molecular detection tests to screen for this neoplasm^5^. However, the Pap test is still essential, since positivity for oncogenic HPV still requires cytological information^6,7^ as a follow-up.

Even with molecular diagnosis, Pap smear is still the screening method used in low-income countries: its replacement by more expensive methods is a distant reality^3^. Even in high-income countries, well-structured implementation of programs using the Pap smear has reduced cervical cancer incidence and mortality rates by up to 65% in the last 40 years^8^.

Despite the global use of Pap smear to detect cervical cancer, it has inherent limitations^9^, including the fact that it is labor intensive, underscoring the need for strategies that generate more accurate results and with lower rates of false-negative, false-positive and unsatisfactory results. Since visual interpretation is time-consuming, subjective and requires highly specialized human interaction^10–15^, increasing efforts over the past few decades have been aimed at developing automated analysis of Pap smear data.

In recent years, machine learning algorithms have been proposed to tackle these challenges. One of the most crucial requirements for an automated system powered by computer vision and machine learning techniques is a collection of hundreds (if not thousands) of high-quality, well-curated digitized images of Pap smears and related metadata. The existing FAIR (Findable, Accessible, Interoperable, and Reusable)^16^ data about Pap smears is limited, and without representation of cells with pre-neoplastic alteration of all classes, mostly composed of cut-out cells, synthetic, non-standardized images, and from liquid-based cytology^17–23^.

The most widely used FAIR data in Pap smear image classification studies for cervical lesion detection is the Herlev base^5,17,21,22,24^. This database comprises a total of 917 images: each image contains a single cervical cell and is assigned to one of seven classes of a pre-neoplasic lesion. Other FAIR data used in Pap smear image classification studies include the SIPaKMeD^25^ database, with 4,049 cells (in 966 images) assigned to one of five categories of cell types, with no pre-neoplastic alteration.

Another issue with most of the databases, including Herlev and SIPaKMeD, is that they are not classified according to the Bethesda System nomenclature, created in 1988 to standardize the terminology of cervicovaginal cytology in order to reduce high variability in the communication of results. The Bethesda System is considered the most used, uniform and reproducible terminology among different pathologists and laboratories^26,27^.

**Fig. 1:**
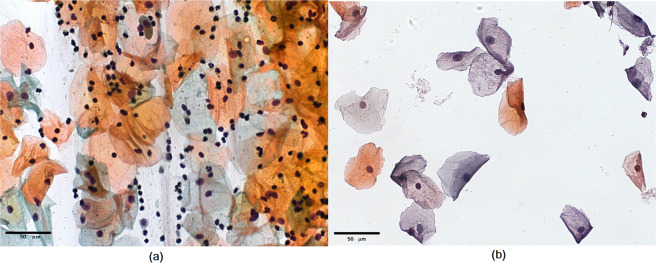
Illustration of (**a**) conventional cytology and (**b**) liquid-based cytology.

A further complication is that many previous works have relied on image collections with cut-out cells and/or unrealistically “clean” images, similar to those in liquid-based cytology^10,20,25,28^. Figure 1 illustrates the drastic difference between images acquired using the conventional and the liquid-based cytology. Conventional cytology has overlapping cells, leukocytes, red blood cells, and mucus, and the image is hugely varied and significantly more complex for analysis than that of liquid-based cytology, which presents homogeneity in cell distribution and absence of obscuring factors^29^.

Constructing and delivering a database of real images of conventional cytology from Pap smear will have considerable impact on computer vision and machine learning methods for knowledge discovery. It allows the comparison of existing algorithms, as well as the investigation of new prediction methodologies that are much more realistic from a biological perspective, and necessary to make such methods valuable to routine cervical cancer screening. Therefore, there are two major challenges: algorithms that allow the identification and classification of cells in a precise way, and, a complete, representative database with accurate information that enables the development/training of automatic methodologies to support the professional’s decision.

**Fig. 2:**
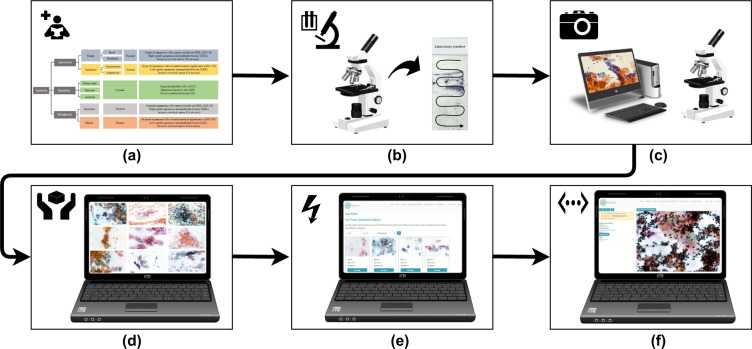
CRIC Database workflow. (**a**) Latest protocols for taxonomy; (**b**) microscope screening for smear selection; (**c**) photo-documentation of smears; (**d**) image selection and curation; (**e**) insertion of images in CRIC, and (**f**) manual classification of cells by cytopathologists at CRIC.

This article presents a web platform, and uses this platform to support the development of a new cervical cell database, “CRIC Cervix”, a collection of images obtained from conventional Pap smear. The Center for Recognition and Inspection of Cells (“CRIC”) is a collaborative consortium among researchers that aims to provide cell collections to the scientific community. The CRIC Cervix images are similar to those obtained during an examination, with many cells per image and 150 dpi resolution. Cytopathologists classified the cells following the protocol defined by the Bethesda System nomenclature. In addition to the cells being individually identified and classified manually by different specialists, the full image field of collected cells also has a diagnosis. The information is available on the CRIC Searchable Image Database web platform, https://database.cric.com.br, which provides a publicly available web tool with information about cervical cells. The goal of this platform, as illustrated in Fig. 2, is to enable the community to explore cell morphology and variability, and facilitate discoveries from the image collections.

## Results

### Database contents

The collection CRIC Cervix has 400 images of Pap smears and 11,534 classified cells. In addition to https://database.cric.com.br, all the images in the current release are available in figshare^30^ as a collection, 10.6084/m9.figshare.c.4960286.v2, under a Creative Commons Attribution 4.0 International license. The classification of the cells is provided in a CSV and JSON file formats with a companion README file that provides human readable details of CSV headers and JSON fields.

The collection is the first to cover conventional cytology cervical cells with classifications performed by several cytopathologists based on the Bethesda System nomenclature, which is the standardized terminology and most used worldwide in the area of cervical cytopathology. The cells in the CRIC Cervix collection are classified in six (6) classes: (1) negative for intraepithelial lesion or malignancy (NILM); (2) atypical squamous cells of undetermined significance, possibly non-neoplastic (ASC-US); (3) low-grade squamous intraepithelial lesion (LSIL); (4) atypical squamous cells, cannot exclude a high-grade lesion (ASC-H); (5) high-grade squamous intraepithelial lesion (HSIL); and (6) squamous cell carcinoma (SCC).

Another unique contribution of the proposed database is that it contains real images of conventional cytology with a variable number of cells, for example, including cells that overlap, which is often present in a smear of cytology, and must be considered when designing and testing automated cytology screening^23,31^. A list of differences among the cervical cell databases are listed in Tables 1 and 2.

**Table 1:** Comparison of properties among databases.

Property	CRIC Cervix	Herlev	SIPaKMeD
Number of images	400	917	966
Cells per image	Variable	1	Variable
Image size (in pixels)	1,376 × 1,020	Variable	2,048 × 1,536
Resolution	0.228 *μ*m/pixel	0.201 *μ*m/pixel	Unknown
Classification	Manual	Manual	Manual
Classified cells	11,534	917	4,049
Validation	3 cytopathologists	2 cyto-technicians	expert cytopathologists
Download Page	database.cric.com.br	mde-lab.aegean.gr/downloads	www.cs.uoi.gr/~marina/sipakmed.html

**Table 2:** Number of cells per characteristics in databases.

Cell type	Cell count		
	CRIC Cervix	Herlev	SIPaKMed
NILM	6,779	144 (*)	(***)
ASC-US	606	0	(***)
ASC-H	925		(***)
LSIL	1,360	182	(***)
HSIL	1,703	493 (**)	(***)
SCC	161	0	(***)
**Total**	**11,534**	**819**	**4,049** (***)

(*) Small requirements and Intermediate squamous epithelial.

(**) Intermediate squamous epithelial and Severe squamous non-keratinizing dysplasia.

(***) Cell categories cannot be translated into the Bethesda System nomenclature.

Both in the total number of cells and the number of cells per class of lesion, CRIC Cervix is the largest (11,534) database available for real cancer cells, providing a broad diversity of neoplastic lesions to date. Also, the CRIC images contain many of challenges often found in ordinary exams, including overlapping cells and inflammatory cells. Classifications are performed by cytopathologists, using the standardized Bethesda System nomenclature. Although the Herlev database has more images, it contains far less cells. As for the Sipakmed database, it has isolated cells from Pap smear slides, and additionally uses a non-Bethesda-conforming classification, dividing cells into five categories: superficial-intermediate cells, parabasal cells, koilocytotic cells, dyskeratotic cells, and metaplastic cells, including normal, abnormal, and benign cases.

### Web platform

In this work, we implemented the CRIC Searchable Image Database, available at https://database.cric.com.br, allowing the user to browse the images via a modern web-interface, easing data access and acceleration of discoveries (Supplementary Video). In addition to the web-interface, the platform is also accessible by a REST (Representational State Transfer) API that makes it possible to add interoperability with third-party projects. The CRIC Searchable Image Database backend uses several technologies, including the web framework Express^32^ for Node.js^33^, which supports the view system, routing, middleware, and other capabilities. In order to connect the web framework with the data, CRIC uses Sequelize^34^ which offers Object Relational Mapping (ORM) objects to the relational database systems, here enabled by MySQL^35^. The front-end uses Angular^36^, which is a JavaScript framework for building web apps. The code is available at https://github.com/CRICDatabase/searchable-image-database and it is released under an open source license that allows anyone to reuse it, particularly if considering the documentation, which is available at https://cric-database.stoplight.io/docs/searchable-image-database-nodejs/reference/cric.v2.yaml.

**Fig. 3:**
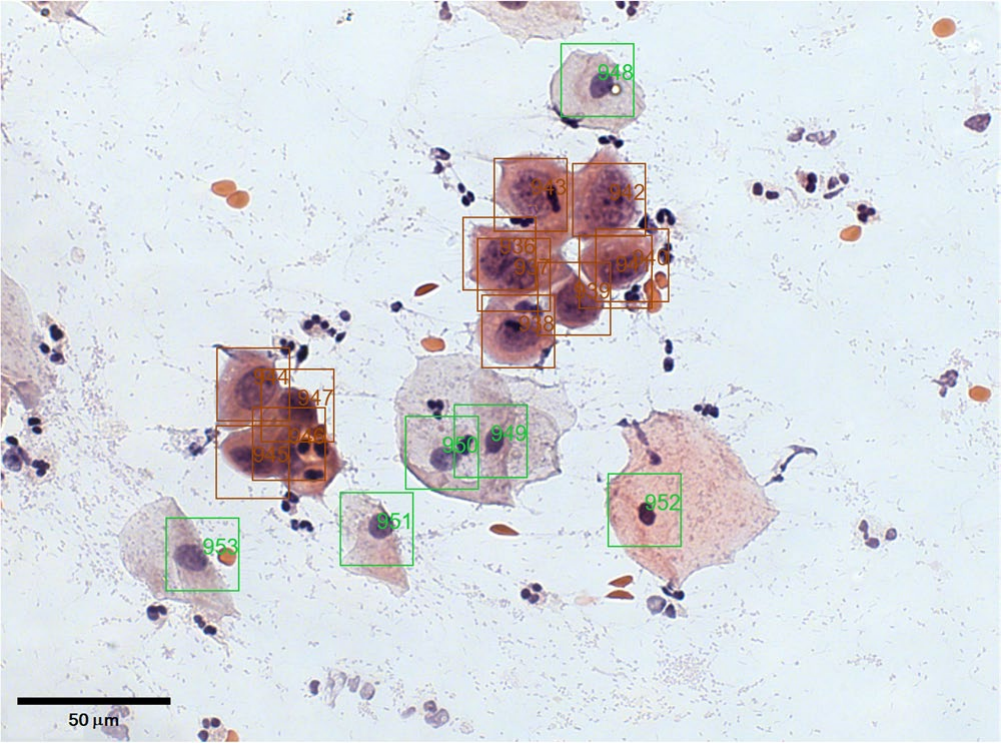
CRIC Cervix Microscope Slide Image #383 with annotations.

After an image is uploaded to the CRIC Searchable Image Database, the user can identify and classify cells manually. The interface captures the coordinate of the click performed by the user, prompts the user for the cell classification, saves the information, and updates the image in the user’s web browser with a square around the latest classified cell. The end result is a curated image such as Fig. 3. In addition to individual cell information, each image has a diagnosis following the most severe case among the classified cells in the image. The user can download the raw images, augmented images (such as Fig. 3), and associated data of the collection.

**Fig. 4:**
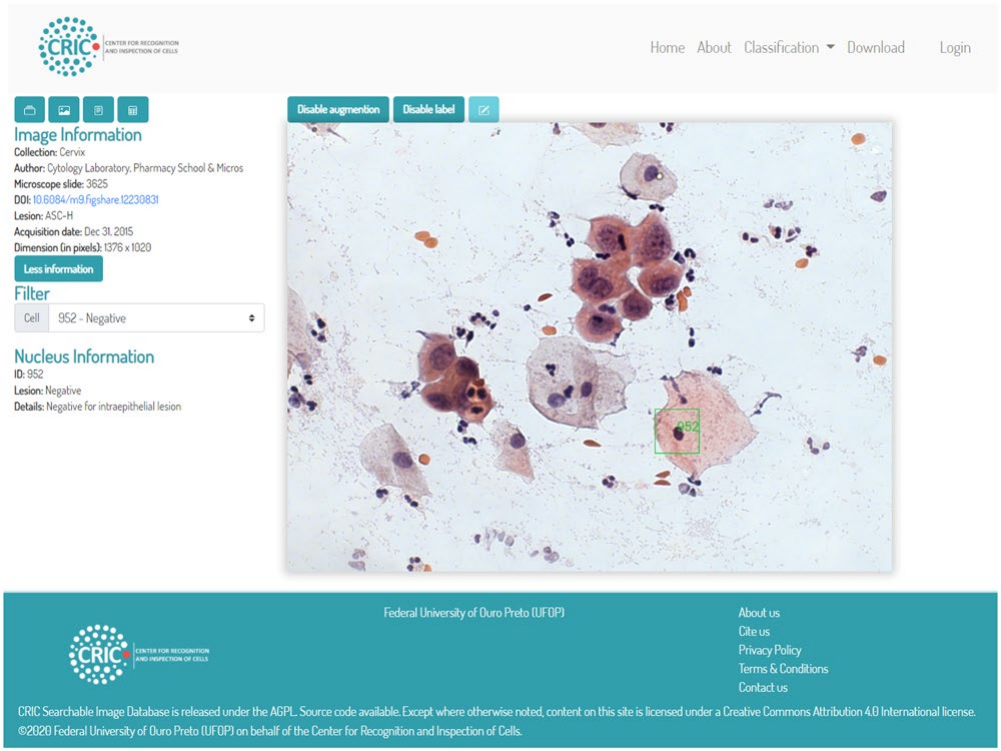
CRIC Searchable Image Database currently available.

Figure 4 illustrates the platform dashboard where the user has an augmented view of the images with squares around the cells. If the user owns the image, they can add or edit the classification of the cells. We provided a playground area available at https://playground.database.cric.com.br/ where users may login to their own area to upload and classify their images. In the image upload, one must provide information about the captured data, author, a reference number from your laboratory, a DOI, if available, as seen at Fig. 4.

At the classification page, the user may access all the images of CRIC Cervix collection. They are presented in a reduced version with information on the slide, final diagnostic, and the number of cells in the image. When accessed in detail, the image is shown with the classification markings that may or may not be enabled. Lesions can also be accessed individually. The images can be saved, with or without the classification labels, and the labels can also be saved in JSON or CVS format. The files contain information on the lesion’s classification and the position (*x, y*) in the image where it occurs. The markings were made in such a way as to be located inside the nucleus of the cell that contains the lesion.

At the download page, the user can save the entire collection, images, and classifications simultaneously or separately.

## Discussion

We introduced the CRIC Searchable Image Database platform, which includes CRIC Cervix, a collection of cell images that are publicly available. This dataset of real images of conventional cervical cytology also have corresponding manual classification of pre-neoplastic cell lesions following the Bethesda System, and performed and certified by cytopathologists. The database will contribute significantly to the development of methodologies and/or automated products in computer-assisted cytology. It will also benefit researchers in machine learning who need labeled data for testing algorithm performance, particularly those related to deep convolutional neural networks^37^.

The use of the CRIC platform and collections will allow discoveries regarding computational and biological descriptors that influence the decision about the presence of a lesion, supporting creation of new hypotheses about the differentiation of low and high grade cell lesions. We expect to further the development of image processing and computer analysis tools for the Pap smear, which are likely to augment the efficiency of visual inspection carried out by cytopathologists. Portraying the slide elements quantitatively allows the increase of the reliability and quality of the examination results. The goal is to reduce the false negatives, minimizing the number of lesions that are not detected or go unnoticed by professionals, providing additional information to support the decision of the specialist in cytopathology in order to obtain more accurate results.

CRIC Cervix has been used by the CRIC research group in several efforts. Araujo *et al*.^38^ introduced a fast way for image ranking from large datasets using convolutional neural networks available in a tool named pyCBIR. They also developed a segmentation algorithm that applies a convolutional neural network trained with patch-images to identify and rank abnormal cell regions^39^. More recently, they presented an analysis framework, CRIC-feat, which streamlines the investigation of different image databases and respective descriptors, particularly applicable to Pap images^40^. Isidoro *et al*.^41^, developed a methodology for automatic classification of cervical cell samples based on non-geometric characteristics present in cell nuclei, excluding the use of segmentation. Such studies can feasibly be explored in different ways by other researchers when the database becomes available, including new segmentation and cell classification methodologies.

An additional impact of the CRIC Searchable Image Database platform is an open-source web application for cell center annotation with single mouse-click and augmented view of the images with the cell annotation. The CRIC platform targets individual users or small groups without the financial resources to use commercially available digital histopathology software or the technical expertise to use complex and opaque solutions^42,43^.

Besides commercially available digital histopathology software, e.g. provided by the manufacturers of the scanner hardware, there are many open source software solutions available for slide viewing and analysis, with some products supporting whole-slide images and others using standard graphics formats. Many solutions provide not only annotation capabilities, but also plug-in systems for automated analysis or pre-processing, e.g. Icy^44^, CellProfiler^45^ or SlideRunner^43^. However, none of the previous mentioned tools allow their users to publish the images to the general public.

The CRIC platform allows researchers to define collections, upload images they have, annotate cells in their images, share their images and associated annotations, and download the data to be used in computer vision and machine learning experiments.

At the moment, the CRIC Cervix collection includes normal squamous cells with precursor and invasive lesions. Updates will include normal glandular cells with precursor and invasive lesions. The authors opted for this strategy due to the fact that among invasive cervical cancers, the most common in the population is squamous cell carcinoma, which account for 75–90% of all cases, depending on the study^46–52^.

Finally, the platform updates will increase the cytology collections and add more functionality at different user access levels, including manual segmentation of cervical cells. The CRIC Searchable Image platform also can support cytology research with other cell types, and we expect to include anal cytology soon.

## Methods

The Research Ethics Committee approved this work at the Federal University of Ouro Preto, Minas Gerais, Brazil, through the document with protocol number 1944523. Due to limitations imposed by such a document, it is not possible to provide demographic information on the participants that contributed to the image dataset, including age and the ethnic diversity. This limitation exists because the participants did not provide consent for data sharing, and the requirement for informed consent was waived by the Research Ethics Committee under the agreements described above.

The Pap smear samples were obtained from female patients from the Southeast region of Brazil, South America. The Pap smear samples were processed and analyzed in the Cytology Laboratory of the Pharmacy School, Federal University of Ouro Preto, Minas Gerais, Brazil. Since the liquid-based cytology method is more expensive, and unavailable to this patient cohort, we employed the conventional cytology method for cytopathological smears, which is recommended by the Brazilian Ministry of Health. The conventional Pap test consists of introducing a speculum into the vaginal canal for exposure and inspection of the uterine cervix. The Ayre spatula collects cells from the ectocervix and the endocervical brush from the endocervix. The collected material was dispensed on a matte tip blade, previously identified in pencil with the patient’s initials and date of birth. In order to preserve the cell structure, the material was fixed in 96% alcohol, and placed in slide bottles supplied by the Cytology Laboratory. These slide bottles were placed in plastic bags, sealed, and transported to the Cytology Laboratory for the processing, and analysis.

In the Cytology Laboratory, a pre-analytical evaluation was carried out, which consisted of selecting the smears that were in adequate conditions for the analysis, and were duly identified with the laboratory registration number. Papanicolaou staining was used, consisting of a nuclear dye, hematoxylin, and two cytoplasmic stains, Orange G and EA 36 or EA 50. Leica AutoStainer XL was used for automatic staining. Then, the smears were assembled automatically, with Entellan between the slide and the coverslip, using the Leica CV5030 equipment. The microscopic analysis, which includes the observation and evaluation of cervical cells under an optical microscope to classify cervical cancer precursor lesions, was performed by a team of 3 cytopathologists. Since 2013, these professionals conduct cytopathological exams for the Brazilian Unified Health System, also known as SUS, and since 2014, they have been carrying out External Quality Monitoring of laboratories that provide services to the SUS in the state of Minas Gerais, Brazil. The entire process was carried out following the Brazilian Ministry of Health recommendation^53^.

The Cytology Laboratory performs Internal Quality Monitoring, which are regular actions to guarantee the quality of the Pap smear, including control of the receipt, assembly and coloration of the samples, use of the 100% Rapid Review method (negative smears are reviewed) and Rapid Pre-scrutiny (scrutiny prior to routine reading), implementation of corrective actions, recording of results, promotion of permanent education to professionals and internal audit^54^.

The first step in the development of the CRIC web platform was the survey of the most recent protocols for taxonomy and the elaboration of a diagram with cell types and respective lesions for guidance in the selection of Pap smears, which served as an interface model used to create the decision tree on the platform.

The smears selected for photo-documentation come from the collection of cervical-vaginal smears obtained through conventional cytology performed at the Cytology Laboratory as part of the routine care. The collection was curated by analyzing the database to access the results of the exam. From the result, the smears were scrutinized under an optical microscope by the three cytologists in order to assess the cytomorphological criteria that best represented each cytological alteration. After consensus among the three specialists, 118 smears from 118 patients were selected. These 118 patients are diagnosed with 3 NILM, 45 ASC-US, 45 LSIL, 12 ASC-H, 10 HSIL and 3 SCC.

The smear photo-documentation was performed by conventional bright field microscopy with a 40× objective and a 10× eyepiece, using a Zeiss AxionCam MRc digital camera coupled to the Zeiss AxioImager.Z2 microscope, with the Axion Vision Zeiss Software, which are at the Multi-User Laboratory of the Biological Sciences Research Center (NUPEB), Federal University of Ouro Preto, Minas Gerais, Brazil. After the photo-documentation, all images obtained by the three cytologists were analyzed and curated, and 400 images were selected to compose the CRIC Cervix dataset.

The classification of cervical precursor and invasive lesions was performed within the CRIC Searchable Image platform, https://database.cric.com.br, according to the Brazilian Nomenclature for cervical cytopathological reports, based on the Bethesda System nomenclature^27^. The classification was carried out by three members of the Cytology Laboratory team, cytologists with experience of 6, 11 and 20 years working in diagnostic cytology.

Each cell was classified by selecting the class corresponding to the lesion, based on morphological criteria prescribed by the Bethesda System nomenclature, followed by marking the center of the cell nucleus. This procedure repeated until all the cells in the image were labeled. The classification protocol followed by the three cytologists started with an independent classification made by an initial professional, then the second specialist checked the labels. Next, the third cytologist performed a review of the markings and if the three answers were in agreement, he/she approved the label. Otherwise, a consensus was reached among the three cytologists to define the final label. After manual labeling, computational routines for pre-processing removed labeled cells near the borders and whose nucleus or cytoplasm structures were not fully visible. This process aims to avoid feeding algorithms with incorrect information about the nucleus or cytoplasm.

## Supplementary information

Supplementary Video

## Data Availability

The collection CRIC Cervix data described in this paper is available at https://database.cric.com.br, and also at 10.6084/m9.figshare.c.4960286.v2^30^.

## Code availability

The classifications where made using https://database.cric.com.br. The source code of the website is available on GitHub, https://github.com/CRICDatabase/searchable-image-database, under GNU Affero General Public License v3.0.
